# Stochastic Leader Gravitational Search Algorithm for Enhanced Adaptive Beamforming Technique

**DOI:** 10.1371/journal.pone.0140526

**Published:** 2015-11-09

**Authors:** Soodabeh Darzi, Mohammad Tariqul Islam, Sieh Kiong Tiong, Salehin Kibria, Mandeep Singh

**Affiliations:** 1 Center for Space Science (ANGKASA), Universiti Kebangsaan Malaysia, Selangor, Malaysia; 2 Department of Electrical, Electronic & Systems Engineering, Universiti Kebangsaan Malaysia, Selangor, Malaysia; 3 Center of System and Machine Intelligence, College of Engineering, Universiti Tenaga Nasional, Selangor, Malaysia; Beihang University, CHINA

## Abstract

In this paper, stochastic leader gravitational search algorithm (SL-GSA) based on randomized *k* is proposed. Standard GSA (SGSA) utilizes the best agents without any randomization, thus it is more prone to converge at suboptimal results. Initially, the new approach randomly choses *k* agents from the set of all agents to improve the global search ability. Gradually, the set of agents is reduced by eliminating the agents with the poorest performances to allow rapid convergence. The performance of the SL-GSA was analyzed for six well-known benchmark functions, and the results are compared with SGSA and some of its variants. Furthermore, the SL-GSA is applied to minimum variance distortionless response (MVDR) beamforming technique to ensure compatibility with real world optimization problems. The proposed algorithm demonstrates superior convergence rate and quality of solution for both real world problems and benchmark functions compared to original algorithm and other recent variants of SGSA.

## Introduction

Increasing interference due to multiple users and other signal sources is one of the fundamental problems in wireless communication and has been extensively studied for many years. Smart antenna systems decrease interference by adaptive beamforming techniques like minimum variance distortionless response (MVDR). It is one of the commonly utilized adaptive array beamforming techniques [1], but it is often not able to form nulls towards any nearby interference sources satisfactorily. Consequently, MVDR may lead to significant performance degradation in the case of unexpected interfering signals [2]. It is difficult and time consuming to solve these problems through conventional empirical approach, and sometimes, in the applied cases, is impractical. Recently, the employment of meta-heuristics algorithm has been growing instead of exhaustive and exact procedures in similar applications [3–7].

Consequently, meta-heuristics and exploratory methods need to provide mathematically reliable solution for this complicated class of optimization problems. However, the performance of these algorithms is often unsatisfactory for cases with three or more interference sources due to issues such as premature convergence and lack of sufficient exploration. Several methods are suggested for increasing the search diversity of SGSA, such as increasing the initial number of leaders (initial *kbest*). However, this significantly increases computational complexity of the force equation in GSA without properly addressing the key issue of dominant agents, with large masses, causing premature convergence. Primary reason for the search pattern domination is because agents are allowed to exert a force proportional to their performance and most SGSA variants allow the best agents to consistently influence all agents. Therefore, this paper, suggests a stochastic leader gravitational search algorithm (SL-GSA) to enhance MVDR beamforming performance by preventing premature convergence and improving overall exploration.

Standard gravitational search algorithm (SGSA) [8] was proposed as a global optimization method for computationally complex real world problems. In SGSA, the particles, called agents, move based on Newton’s law of universal gravitation. The search space is represented as an ‘*n*’ dimensional space and the position of each agent is represented by a coordinate vector of length *n*. The mass of these agents are determined based on their fitness. The performance of each agent is calculated using the fitness function and their positions are updated accordingly. All the SGSA search agents (individuals) globally move toward the agents with heavier masses due to their gravitational force. Hence, superior solutions of the problems are represented by the heavier masses. The global search ability and high performance of SGSA in solving several nonlinear functions have been confirmed previously [8].

The balance between exploration and exploitation is critical for heuristic algorithms to achieve robust and reliable performance. In SGSA, this balance is achieved using the time variant linearly decreasing *kbest* parameter, which determines the number of agents that are allowed to exert force on the others in a given iteration. Thus, the parameter *kbest* is initially large and linearly reduced to provide some protection from premature convergence. This technique still allows the optimization process to be heavily influenced by agents with superior fitness resulting in poor exploration properties. As *kbest* agents are chosen based on their current fitness, it allows agents with superior fitness to attract the others towards optimal solutions. Thus, the algorithm is highly dependent on the best performing agents. However, if the *kbest* agents stagnate at a local optimum, the other agents become practically helpless to prevent premature convergence. The SGSA agents gravitate towards ‘*kbest*’ optimum agents. This allows convergence towards superior solutions but also allows the search to stagnate at local optima.

In this paper, SL-GSA randomly selects agents from a gradually reducing set that removes agents with inferior performance based on the adaptive parameter, *γ*. This directly prevents the domination of the search pattern by any individual agent. Thus, SL-GSA is far less likely to stagnate in a local optimum because it randomly ignores the best particles sometimes. This allows more efficient exploration before final convergence. The proposed new parameter, *γ*, prevents selection of the agents with the worse fitness in the later part of the optimization. This, in conjunction with the linear decrease of the parameter *k*, allows SL-GSA to converge faster than SGSA. This is verified by applying the proposed algorithm to six benchmark functions and two case studies of MVDR beamforming technique. High performance of convergence and quality of final solution compared to original algorithm is achieved as discussed in simulation results. The rest of this paper is organized as follows: Section 2 introduces the brief review of SGSA. The proposed SL-GSA is presented in section 3. The basics of adaptive beamforming and the conventional MVDR technique are explained in section 4 and 5, respectively. The testing of the proposed SL-GSA via benchmark functions and the simulation results obtained via SGSA and its variants are reported in section 6. Section 7 shows the incorporation of MVDR in SL-GSA. The efficiency of SL-GSA for different interferences in two case studies is also reported in this section. Finally, Section 8 concludes this investigation.

## Standard Gravitational Search Algorithm

Standard gravitational search algorithm (SGSA) was presented as one of the recent heuristic population search algorithms [8] based on the mass interactions and laws of gravity. This method uses the Newtonian laws of gravitation to govern the motion of virtual agents through a search space. The *i* ^th^ GSA agent is defined as *X*_*i*_ = *(x*_*i*_^*1*^,*…*,*x*_*i*_^*d*^,*…*,*x*_*i*_^*n*^ *)*, for *i* = 1,2,…,*N* that will be evaluated through their masses. In the definition of *X*_*i*_, *N* is the total number of agents, *n* is the total number of dimensions in the search space. These objects move together toward the agents with heavier masses due to gravitational force. The new position and velocity of *i* ^*th*^ agent at iteration *t* along the *d* ^*th*^ dimension will be upgraded as follows:

$$x_{i}{}^{d}(t+1)=x_{i}{}^{d}(t)+v_{i}{}^{d}(t+1)$$ (1)

vid(t+1)=rand×ivid(t)+aid(t) (2)

where *x*_*i*_^*d*^ and *v*_*i*_^*d*^ is position and velocity of *i* ^*th*^ agent at dimension *d* respectively. *rand*_*i*_ augments a randomized characteristic to the search pattern. The acceleration of agent *i* in iteration number *t* by the law of motion, is calculated as below:

$$a_{i}^{d}(t)=\frac{F_{i}^{d}(t)}{M_{i}(t)}$$ (3)

where *M*_*i*_ is the normalized inertial mass of *i* ^th^ agent that evaluated by fitness function. More efficient agents have heavier masses that are calculated using fitness values. The updated inertia masses are as follows:

$$m_{i}(t)=\frac{fit_{i}(t)-worst(t)}{best(t)-worst(t)}$$ (4)

$$M_{i}(t)=\frac{m_{i}(t)}{\overset{N}{\underset{j=1}{\sum }}m_{j}(t)}$$ (5)

where *fit*_*i*_ *(t)* is the fitness value of the *i* ^th^ agent at iteration *t*. *worst (t)* and *best (t)* are the worst and best fitness of population, respectively that are defined as below (for a minimization problem):

$$worst(t)=\underset{j\in \{1,\ldots ,N\}}{\max}fit_{j}(t)$$ (6)

$$best(t)=\underset{j\in \{1,\ldots ,N\}}{\min}fit_{j}(t)$$ (7)

Then, the total force on *i* ^th^ agent in *d* dimension is calculated as below:

$$F_{i}^{d}(t)=\overset{N}{\underset{j=1,j\neq i}{\sum }}rand_{j}F_{ij}^{d}(t)$$ (8)

One way to increase the performance of SGSA is to make a suitable tradeoff between exploitation and exploration. In order to avoid local optimum stagnation, the algorithm needs to emphasize exploration at beginning. However, in later iterations, exploration must fade out and exploitation needs to increase. *kbest* is a time function that is initialized as the total number of agents and linearly decreased to one finally. Thus, at the beginning, all agents apply the force and, at the end, only one agent applies force to the others [8]. Furthermore, this reduces the number of overall forces that need to be summed to calculate the net force. Hence, Eq (8) is modified to the following:

$$F_{i}^{d}(t)=\underset{j\in kbest,j\neq i}{\sum }rand_{j}F_{ij}^{d}(t)$$ (9)

where *rand*_*j*_ is the random value in the interval [0,1] and *F*_*ij*_^*d*^ is force acting from mass *j* on mass *i* at dimension *t* as follows:

$$F_{ij}{}^{d}(t)=G(t)\frac{M_{pi}(t)\times M_{aj}(t)}{R_{i,j}(t)+\varepsilon }\left(x_{j}{}^{d}(t)-x_{i}{}^{d}(t)\right)$$ (10)

where *ε* is the small constant value to prevent division by zero, *R*_*i*,*j*_ *(t)* and *G(t)* are the Euclidean distance between agents *i* and *j* and the gravitational constant respectively as below:

$$R_{i,j}(t)={\left\|X_{i}(t),X_{j}(t)\right\|}_{2}$$ (11)

G(t)=G0×exp(−β×ttmax) (12)

where *t* is the current iteration, *t*_*max*_ is the maximum iteration number, *β* is a constant value. The gravitational constant determines how much influence the agents have on each other. High gravitational constant basically allows larger acceleration. It is exponentially reduced to allow convergence at the end of the optimization process.

The acceleration of the *i* ^th^ agent at dimension *d* and iteration *t* is calculated by the Newtonian motion laws as following:

$$a_{i}^{d}(t)=\underset{j\in kbest,j\neq i}{\sum }rand_{j}G(t)\frac{M_{j}(t)}{R_{i,j}(t)+\varepsilon }\left(x_{j}{}^{d}(t)-x_{i}{}^{d}(t)\right)$$ (13)

## Stochastic Leader Gravitational Search Algorithm

The method of selecting best agents to apply the force on others in SGSA using a linear time based function, *kbest*, is an elegant way to improve the performance of the algorithm by efficiently compromising between the exploration and exploitation properties. However, selecting best agents to be the leaders may cause the algorithm to get trapped in local optima soon after reaching a near optimal region due to adherence to the leaders. In this paper we propose a modification to overcome this drawback by allowing other agents to lead randomly according to their performances. This is done by modifying Eq (9) as follows:

$$F_{i}^{d}(t)=\sum_{j\in LAI}rand_{j}F_{ij}^{d}(t)$$ (14)

where *LAI* is a vector representing the indices of leaders to be selected from a vector of agents sorted in descending order of fitness, at iteration *t* and can be described as follows:

$$LAI\;(t)=[z_{1}(t),z_{2}(t),\ldots ,z_{i}(t),\ldots ,z_{K}(t)]$$ (15)

$$z_{i}(t)=round(1+(N-1)\times rand_{i}\times \gamma (t)),\;i=1,2,\ldots ,K$$ (16)

γ(t)=γinitial+(γfinal−γinitial)×(ttmax),0≤γ≤1,γinitial>γfinal (17)

where *z*_*i*_ *(t)* is the *i* ^th^ leader index number in the vector of agents which are sorted from the best to the worst at iteration *t*. *γ* is an adaptive factor between 0 and 1 determining the range of selection and is linearly adjusted during the search process from an initial value to a final value based on Eq (17); *rand*_*i*_ produces a uniform random number between 0 and 1. In each iteration, the agents are sorted in a vector based on their performances from heavier masses to lighter ones. Then *K* leaders are selected randomly from the vector according to Eq (16). The selection mechanism is designed to allow selection of weaker agents in early iterations to lead others, resulting in improved exploration. As the iterations proceed, the chance of selecting the weaker agents is reduced by Eq (17) and superior agents attract others, which results in enhanced exploitation and convergence behaviour. By this modification the agents will be more active and the chance of getting trapped in local optima is reduced during first few iterations, while not degrading the overall convergence performance.

**Fig 1:**
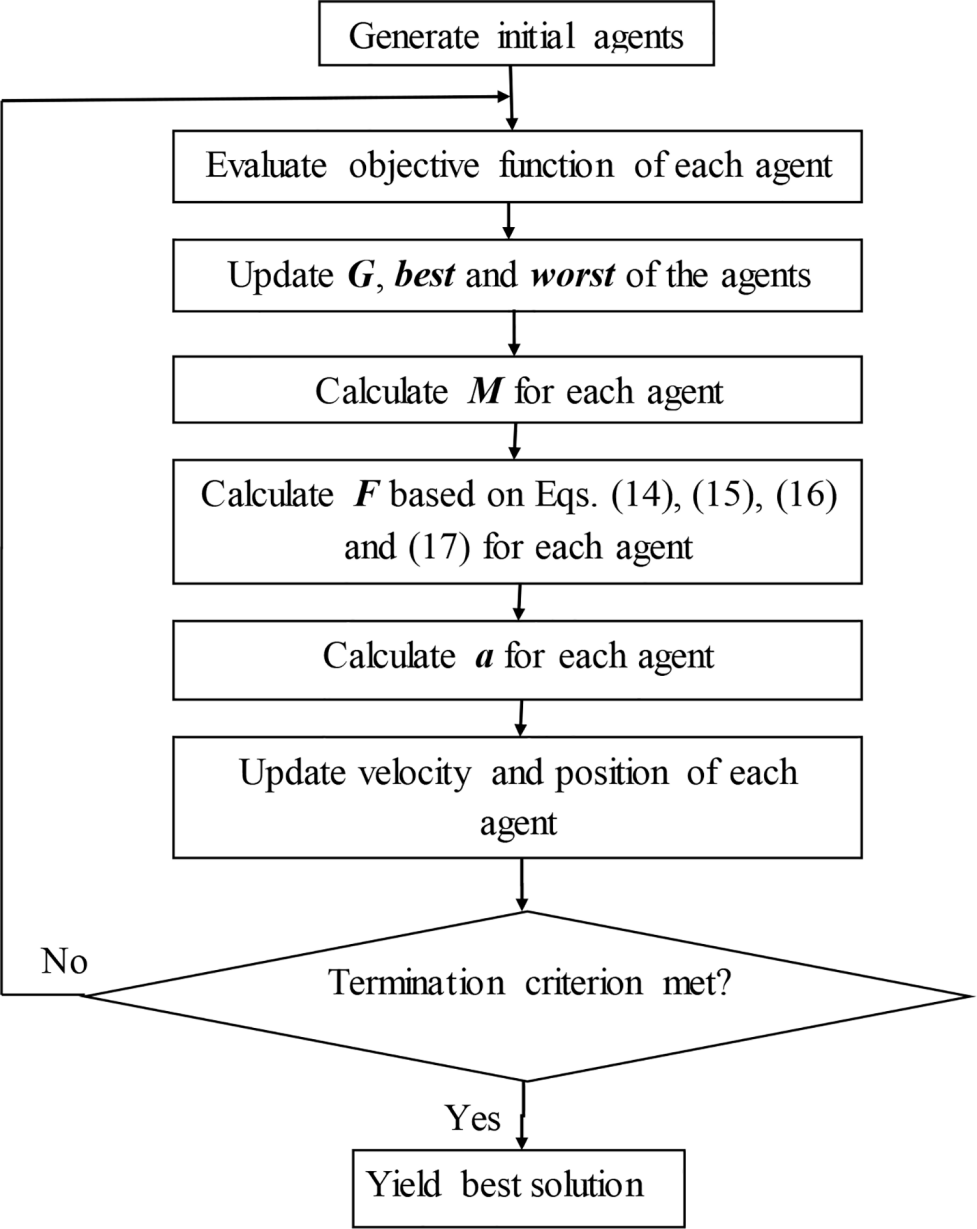
The simplified flowchart of SL-GSA method.

The flowchart for SL-GSA, illustrated in Fig 1, explains the algorithm in detail. It shows that the algorithm is rather convenient and robust as it requires calibration of only one additional parameter, γ.

This modification is designed to improve the early segment of the search process, while allowing the proposed algorithm to behave similarly to SGSA by discriminating against agents with poor performance.

## Adaptive Beamforming

**Fig 2:**
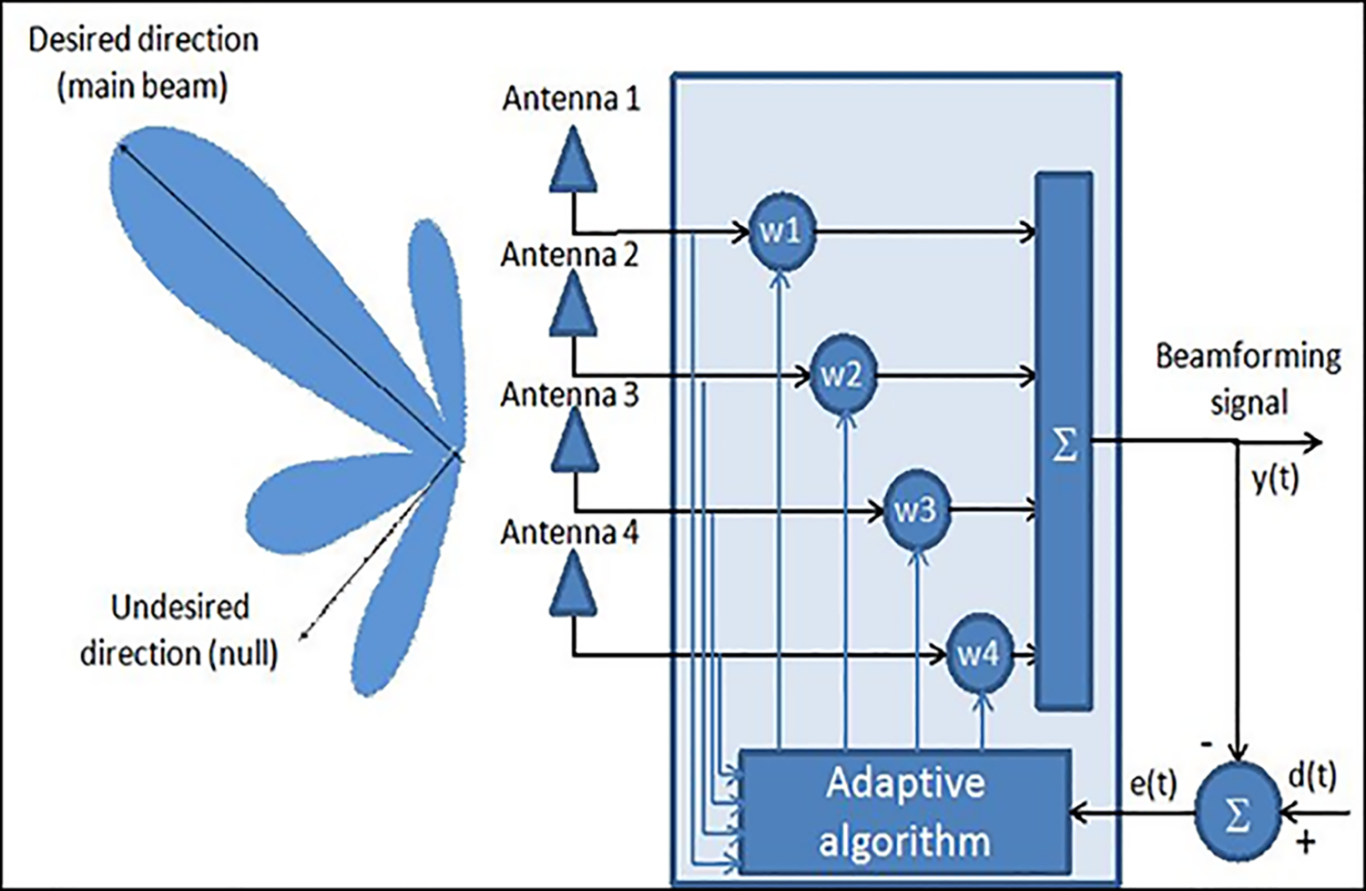
Adaptive beamforming system.

The aim of adaptive beamforming is to steer radiation beam towards the signal of interest (SOI) and produce null towards the interference sources. Beamforming, by signal processing technique, automatically recognizes and adjusts incoming SOIs and interference from received data. Signals from individual antennas were combined linearly after being scaled with the matching weights. This procedure improves the antenna array to obtain maximum gain in the desired signal radiation and nulls in the interference sources. An adaptive algorithm, like MVDR, uses the SOI direction (direction of main beam) and interference directions to produce a linear combination of the signals from each antenna, as illustrated in Fig 2, to achieve optimal signal to interference and noise ratio (SINR) performance. Furthermore, the algorithm must ensure a distortion-less response towards the SOI.

The output *y* is given by a linear combination of the data from the *M* sensors, with signal from each element *x(n)* and weight *w(n)* where * symbolizes the complex conjugate as shown below:

$$y=\sum_{n=0}^{M-1}w_{n}*x_{n}=W^{H}X$$ (18)

$$W^{H}=[w_{0}^{*},w_{1}^{*},\ldots ,w_{M-2}^{*},w_{M-1}^{*}]={\left[W^{T}\right]}^{*}$$ (19)

where *W* is length *N* weights vector, *X* is length *N* received signals vector, and Superscript ^*H*^ is the conjugate transpose (Hermitian vector).

## Conventional MVDR Beamforming

The minimum output energy (MOE) uses as a beamforming technique that implements several optimality standards. MOE has a fixed array gain on desired signal while simultaneously minimizing the output energy. Therefore, when gain is constant, any decline in the energy is because of suppressing interference as below:

$$w_{MOE}=\arg\;\underset{w}{\min}E\{{\left|y\right|}^{2}\},\;w^{H}h_{0}=c$$ (20)

$$\equiv \arg\;\underset{w}{\min}E\{{\left|w^{H}x\right|}^{2}\},\;w^{H}h_{0}=c$$ (21)

where *h*_*0*_ is the steering vector. Lagrange multipliers method can solve this minimization. By solving the gain constraint and using the constraint on the weight, the parameter of Lagrange *λ* can be acquired. The weights can be written as:

$$=c\frac{R^{-1}h_{0}}{h_{0}{}^{H}R^{-1}h_{0}}$$ (22)

where *R* is the covariance matrix, with size of (*M*, 1), for the received signal *x*.

Determining arbitrary constant, *c* = 1, creates the minimum variance distortionless response (MVDR). One of the beamforming techniques presented in literature [1] is known as MVDR due to minimum energy (variance) required for output signal and unity signal gain (desired signal is not distorted). Moreover, the output power that is subjected to a unity gain constraint in the direction of wanted signal must be minimized. The output power can be obtained as below:

$$p=\left\{E{\left|y\right|}^{2}\right\}=E\left\{w^{H}xx^{H}w\right\}=w^{H}E\left\{xx^{H}w\right\}=w^{H}R$$ (23)

MVDR beamforming keeps a distortionless main lobe response towards the desired signal and minimize the array output power simultaneously. The Lagrange multiplier method does not explore the entire search space. Instead, the search is limited to locations identified by Eq (22), where the response towards the SOI is 0 dB. Furthermore, Lagrange multiplier method is reliant on the ‘smoothness’ of the fitness function to achieve satisfactory performance. Thus, this method may have unsatisfactorily low nulling in multiple interference scenarios, which may have significantly adverse effect on any system performance [9]. Hence, the introduction of a meta-heuristic technique with improved global exploration capability is proposed in this paper for beamforming application.

## Model Verification

In order to analyse, compare and validate the effectiveness and efficiency of the proposed SL-GSA, the proposed algorithm is implemented on six standard benchmark functions and the results are compared with SGSA [8], OBGSA [10] and MGSA [11]. These functions and parameters used in this study are summarized in Tables 1 and 2, respectively. The first four functions are unimodal and the other two are multimodal. The results shown in Tables 3, 4 and 5 are recorded from 30 simulation of each algorithm. The worst, mean, median, best and standard deviation are calculated from aforementioned 30 runs and compared.

**Table 1:** Standard benchmark functions.

Test function	Dimension(n)	Range	Optimum
$F_{1}(X)=\sum_{i=1}^{n}{\left(\sum_{j=1}^{i}x_{j}\right)}^{2}$	30	[–100,100]^n^	0
$F_{2}(X)=\sum_{i=1}^{n}ix_{i}^{4}+random[0,1)$	30	[-1.28,1.28]^n^	0
F3(X)=∑i=1n[100(xi+1−xi2)2+(x−i1)2]	30	[–30,30]^n^	0
*F*_4_(*X*) = max{|*x*_*i*_|,1 ≤ *i* ≤ *n*}	30	[–100,100]^n^	0
F5(X)=πn{10sin2(πy)1+∑i=1n−1(yi−1)2[1+10sin2(πyi+1)]+(yn−1)2}+∑i=1nu(xi,10,100,4)yi=1+xi+14,u(xi,a,k,m)={k(xi−a)m......,xi>a0.............,−a<xi<ak(−xi−a)m,xi<−a	30	[–50,50]^n^	0
$F_{6}(X)=-20\exp\left(-0.2\sqrt{\frac{1}{n}\sum_{i=1}^{n}x_{i}{}^{2}}\right)-\exp\left(\frac{1}{n}\sum_{i=1}^{n}\cos(2\pi x_{i})\right)+20+e$	30	[–32,32]^n^	0

**Table 2:** Parameters used in this study.

Parameter	Description	SGSA	MGSA[11]	OBGSA[10]	SL-GSA
*N*	Population size	50	50	50	50
*G*_*0*_	Initial value of gravitational constant	100	100	150	100
*β*	Constant value	20	20	20	20
*ε*	Constant value	2.22×10^−16^	2.22×10^−16^	2.22×10^−16^	2.22×10^−16^
*t*_*max*_	Maximum iteration number	500,1000	500,1000	500	500,1000
*γ*_*final*_	Final Gamma	N/A	N/A	N/A	0.1
*γ*_*initial*_	Initial Gamma	N/A	N/A	N/A	0.6

**Table 3:** Minimization result of benchmark functions in Table 1 with *t*_*max*_ = 500.

Function	Method	Worst	Mean	Median	Best	Standard deviation
F_1_	SGSA [11]	1120.8	486.03	418.38	261.32	189.9
	MGSA [11]	125.9	65.05	60.36	28.011	24.48
	SL-GSA	122.5	51.02	46.92	26.202	23.55
F_2_	SGSA [11]	2.304	0.151	0.0418	0.014	0.421
	MGSA [11]	0.0518	0.0102	0.0082	0.0026	0.0101
	SL-GSA	0.0307	0.0101	0.0101	0.0021	0.0051
F_3_	SGSA [11]	152.59	35.99	27.71	26.28	30.65
	MGSA [11]	29.36	27.23	27.16	25.94	0.644
	SL-GSA	29.09	26.61	26.59	25.20	0.627
F_4_	SGSA [10]	7.34	3.73	3.51	0.086	1.88
	OBGSA [10]	8.56×10^−9^	5.11×10^−9^	4.89×10^−9^	3.36×10^−9^	1.46×10^−9^
	SL-GSA	5.62×10^−9^	3.43×10^−9^	3.42×10^−9^	2.05×10^−9^	8.63×10^−10^
F_5_	SGSA [10]	2.88	0.736	0.374	0.0111	0.946
	OBGSA [10]	0.0099	0.0017	6.24×10^−4^	2.76×10^−5^	0.003
	SL-GSA	3.36×10^−18^	1.43×10^−18^	1.28×10^−18^	6.82×10^−19^	6.18×10^−19^
F_6_	SGSA	2.57	0.47	2.78×10^−4^	8.20×10^−6^	0.69
	SL-GSA	1.64×10^−8^	1.12×10^−8^	1.04×10^−8^	6.79×10^−9^	2.92×10^−9^

**Table 4:** Minimization result of benchmark functions in Table 1 with *t*_*max* =_ 1000.

Function	Method	Worst	Mean	Median	Best	Standard deviation
F_1_	SGSA [11]	530.85	249.76	243.24	67.85	106.14
	MGSA [11]	49.53	27.1	26.22	8.33	10.36
	SL-GSA	41.46	16.04	10.80	7.09	10.12
F_2_	SGSA [11]	0.0433	0.0227	0.022	0.0106	0.0077
	MGSA [11]	0.0229	0.0077	0.0064	0.0026	0.0049
	SL-GSA	0.0188	0.0067	0.0056	0.0022	0.0047
F_3_	SGSA [11]	152.68	32.86	26.1	25.79	26.55
	MGSA [11]	29.36	27.23	27.16	25.94	0.644
	SL-GSA	25.38	25.05	25.12	23.86	0.260
F_4_	SGSA [8]		8.5×10^−6^	3.7×10^−6^	3.7×10^−6^	
	SL-GSA	1.03×10^−9^	1.11×10^−9^	1.12×10^−9^	8.52×10^−10^	1.09×10^−10^
F_5_	SGSA [8]		0.01	4.2×10^−13^	0.01	
	SL-GSA	9.05×10^−19^	5.69×10^−19^	5.72×10^−19^	2.72×10^−19^	1.65×10^−19^
F_6_	SGSA [8]		1.1×10^−5^	6.9×10^−6^	6.9×10^−6^	
	SL-GSA	6.98×10^−9^	6.14×10^−9^	6.24×10^−9^	4.86×10^−9^	6.20×10^−10^

**Table 5:** Comparison of gamma (*γ*) boundaries for minimization of F_6_ with *t*_max = 500_.

*γ*_*initial*_- *γ*_*final*_	Worst	Mean	Median	Best	Standard Deviation
1–0	1.90×10^0^	6.57×10^−1^	6.44×10^−1^	5.28×10^−9^	0.670
0.9–0.5	0.0711	0.0046	6.73×10^−8^	3.76×10^−8^	0.0163
0.9–0.1	2.59×10^−8^	1.37×10^−8^	1.45×10^−8^	8.32×10^−9^	4.40×10^−9^
0.8–0.4	6.61×10^−8^	3.56×10^−8^	3.17×10^−8^	1.86×10^−8^	1.18×10^−8^
0.6–0.1	1.64×10^−8^	1.12×10^−8^	1.04×10^−8^	6.79×10^−9^	2.92×10^−9^

These benchmark functions provide an efficient method for comparison between heuristic algorithms along with providing insight into specific aspects such as rate of convergence and stability of the algorithm. The parameters presented in Table 2 are chosen to conduct a fair comparison between different variants of SGSA.

Tables 3 and 4 compare the optimization performance of the proposed algorithm to the results reported in literature for 500 and 1000 iterations, respectively.

**Fig 3:**
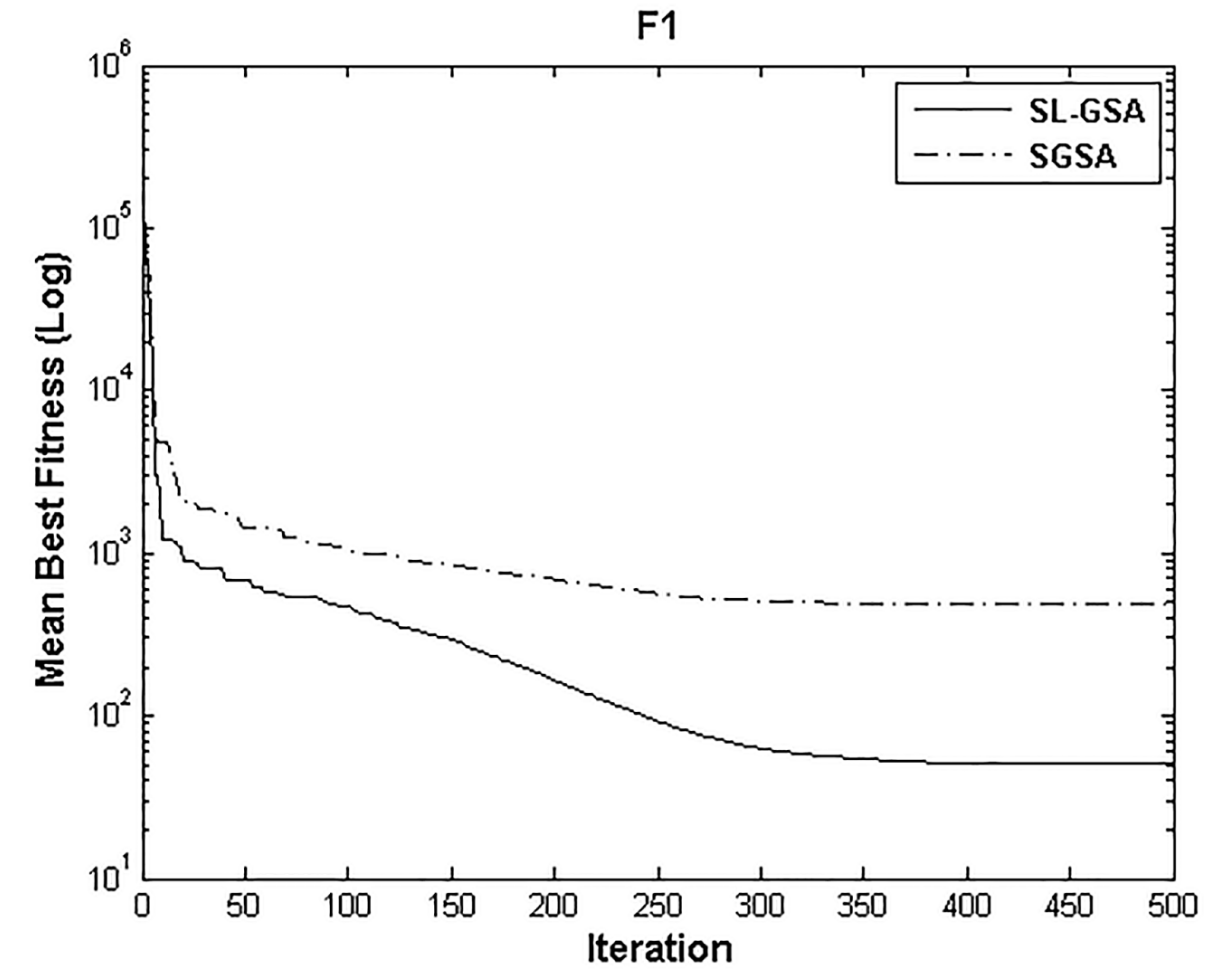
Performance comparison of SGSA and SL-GSA for minimization of F_1._

**Fig 4:**
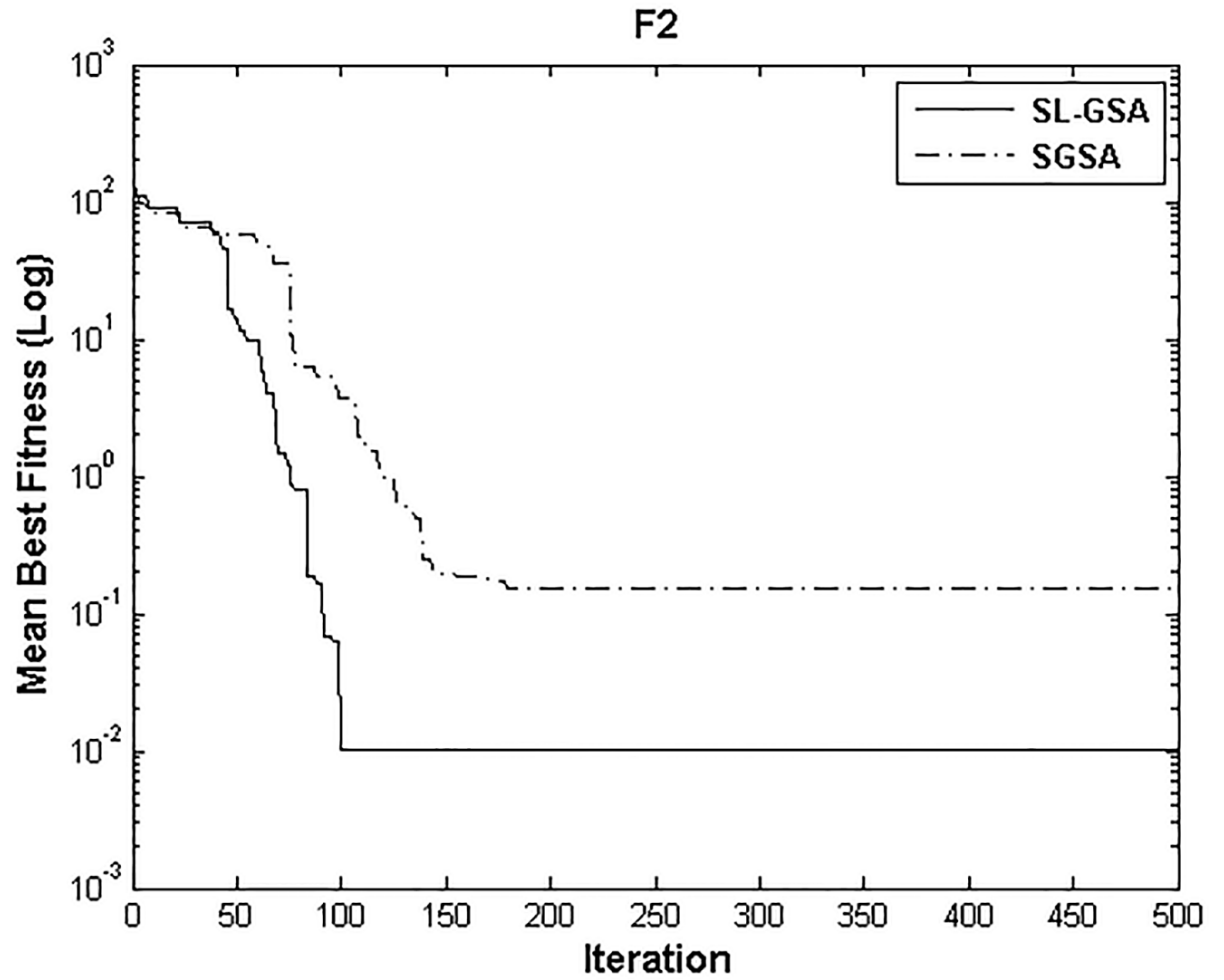
Performance comparison of SGSA and SL-GSA for minimization of F_2._

**Fig 5:**
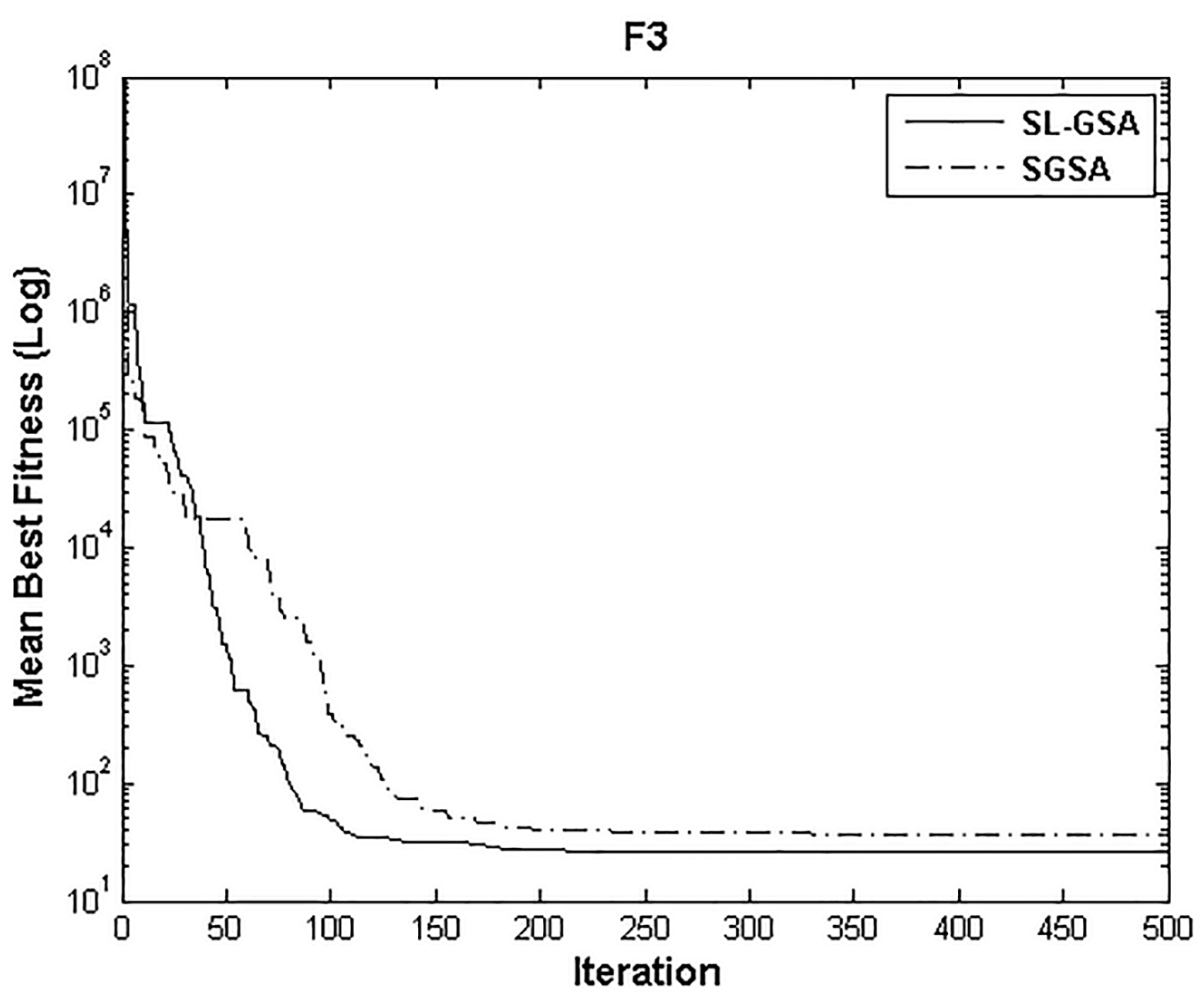
Performance comparison of SGSA and SL-GSA for minimization of F_3._

**Fig 6:**
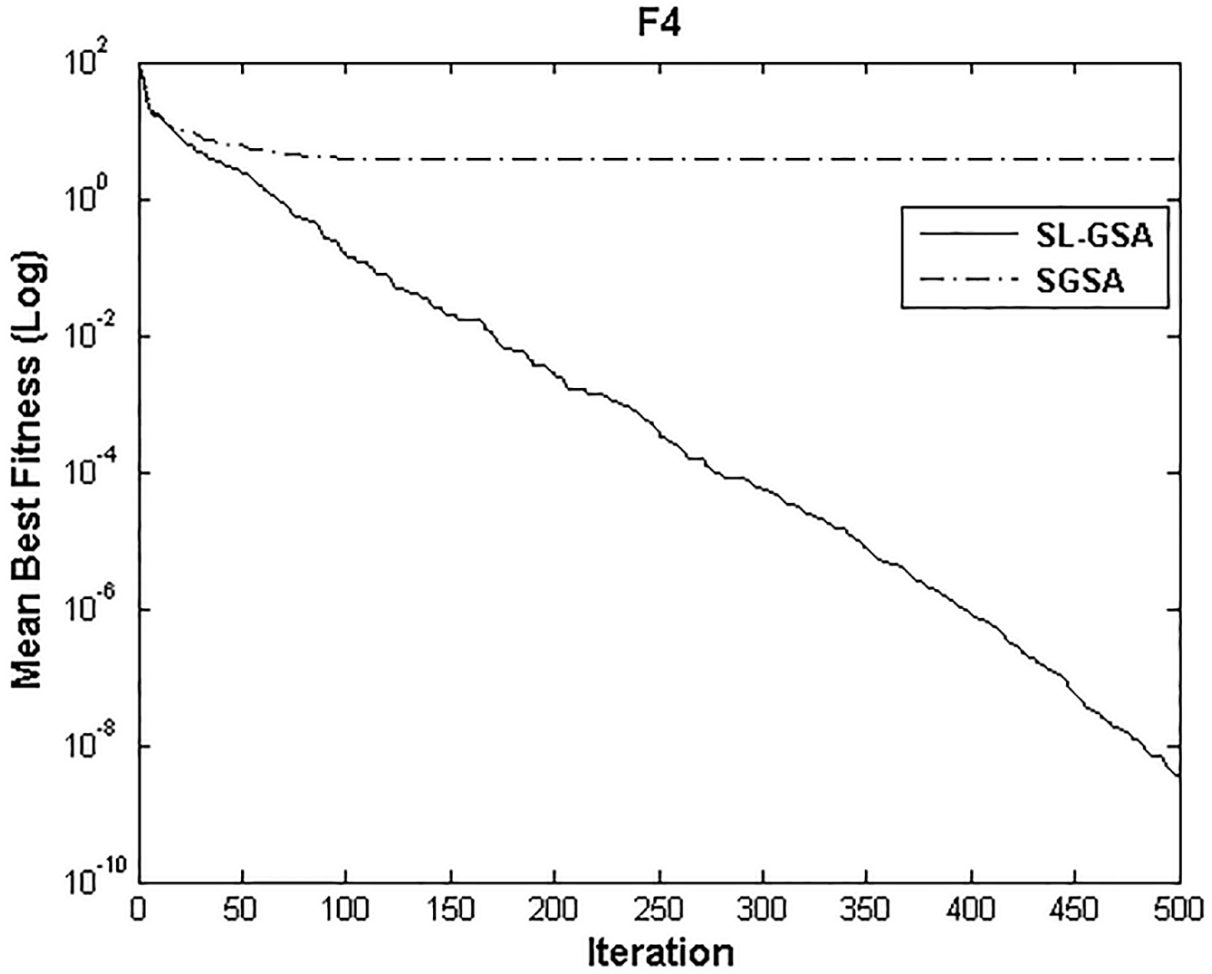
Performance comparison of SGSA and SL-GSA for minimization of F_4._

**Fig 7:**
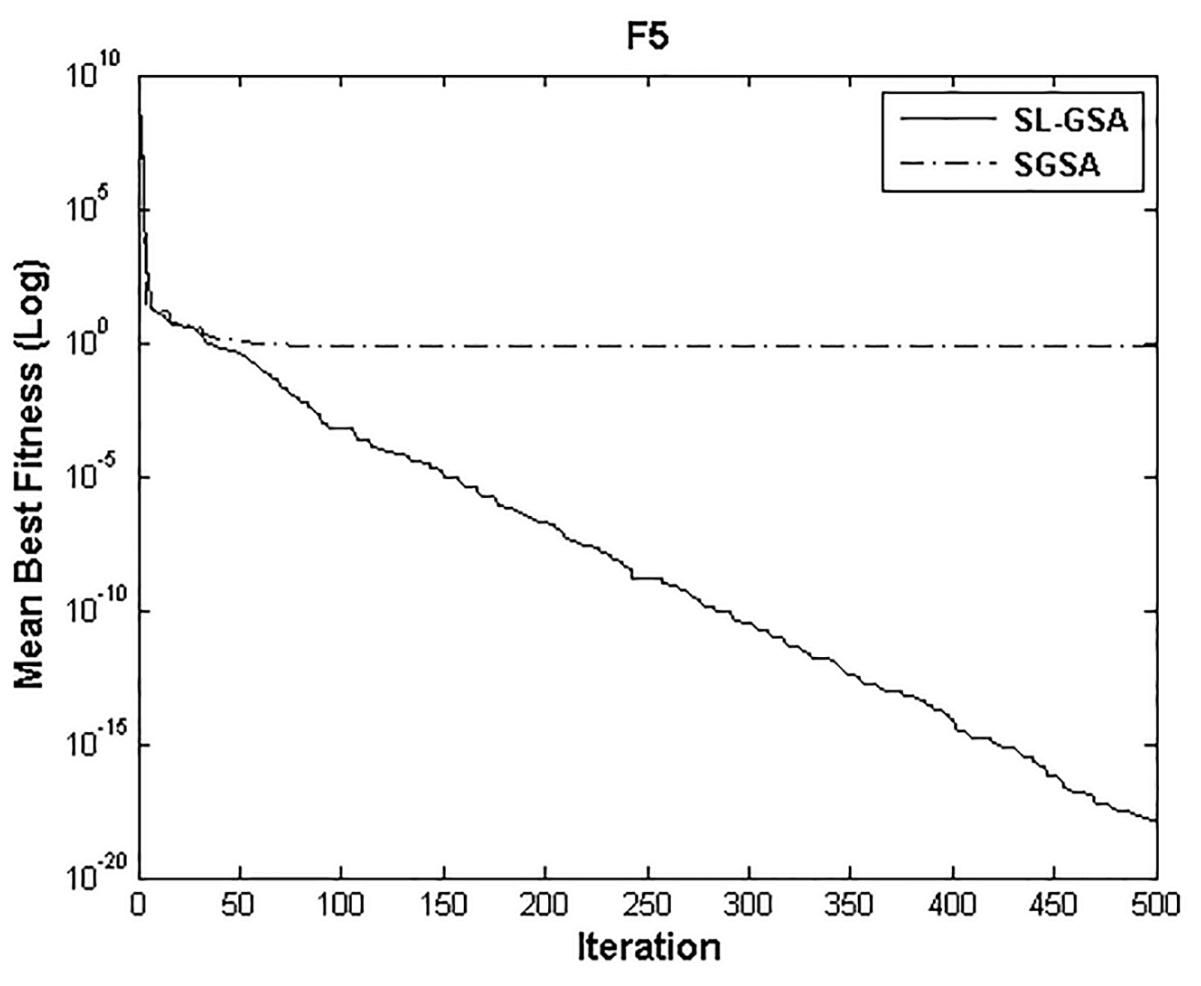
Performance comparison of SGSA and SL-GSA for minimization of F_5._

**Fig 8:**
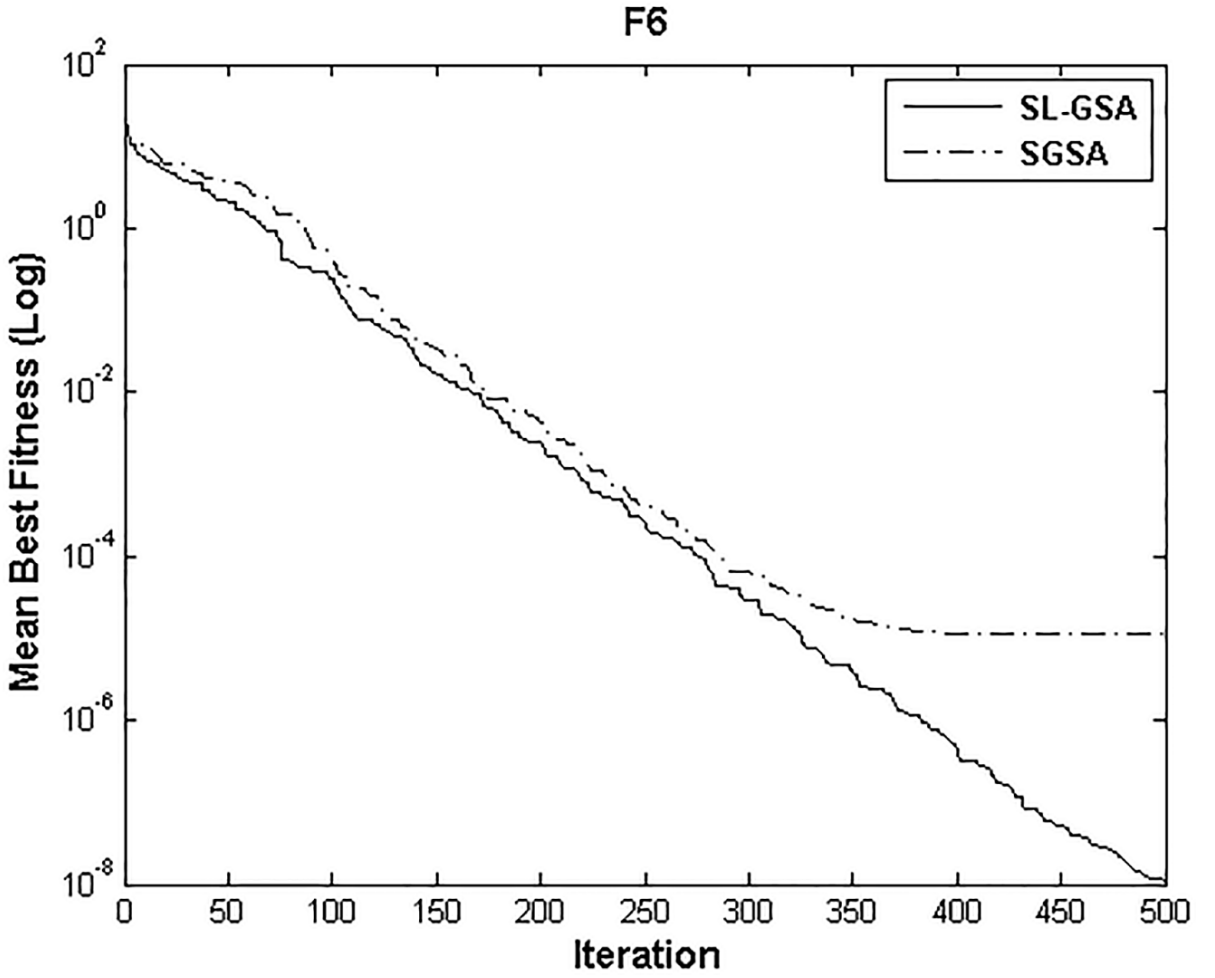
Performance comparison of SGSA and SL-GSA for minimization of F_6._

According to the results of Tables 3 and 4, proposed SL-GSA provides considerably lower mean fitness values in 500 iterations than SGSA in 1000 iterations for all functions. For the functions F_1_, F_2_, and F_3_, proposed algorithm also provides better results in 500 iterations compared with MGSA [11] at equal number of iterations. Furthermore, for the same number of iterations, results achieved by proposed SL-GSA are better than those calculated by OBGSA [10] for the functions F_4_ and F_5_. The comparison for 1000 iterations of SGSA and SL-GSA on F_6_ concludes the comparisons with a prevalent trend of superior SL-GSA performance. Moreover, the standard deviations for 30 runs of proposed algorithm are generally smaller than those of SGSA, OBGSA and MGSA for both 500 and 1000 iterations, which strongly indicate that SL-GSA is significantly more stable and can produce better solutions more consistently. The results indicate, for all tested functions, the mean fitness values achieved by proposed algorithm are significantly lower than SGSA, OBGSA and MGSA, in same number of iterations. Therefore, the results of Tables 3 and 4 establish the proposed SL-GSA as more robust and capable than SGSA and its variants. The convergence histories of the mean best fitness of SGSA and SL-GSA in the 30 independent runs for six benchmark functions are shown in Figs 3–8. The Figs show that the convergence rate of the proposed SL-GSA is better than SGSA in 500 iterations.

Figs 3–8 show that the SL-GSA has faster varying curves of mean best fitness than SGSA and achieves lower and better solution compared with SGSA. Furthermore, Figs 6 and 7 indicate that SGSA suffers from premature convergence during optimization of F_4_ and F_5_ respectively. They stopped improving approximately before 50 iterations and hence produced an inferior solution. The overall results show that SL-GSA performs significantly better than SGSA for both unimodal and multimodal optimization problems consistently. The difference in exploration performance in the early phase between SGSA and proposed SL-GSA is vividly visible in most cases as the SL-GSA curve falls away from the SGSA before the hundredth iteration. This early advantage translates into further superior performance in the later phase of exploitation as the SL-GSA graph continues to separate further from the SGSA curve in case of F_1_, F_4_, F_5_ and F_6_.

### 6.1 Sensitivity Analysis of *γ* Parameters

The *γ* parameters provide the distinctive characteristics to the proposed algorithm. Thus, in this section, the effects of the *γ* parameters on the proposed algorithm’s performance are analyzed in detail. The *γ*_*initial*_ determines the performance cutoff for eligible agents at the beginning of the optimization. The value 0.6 roughly corresponds to the top 60 percentile in terms of performance. This cutoff criterion becomes stricter with progressive iterations. As *γ* decreases linearly towards the *γ*_*final*_ value of 0.1, the agents must be within the approximately top 10 percentile to be eligible for selection. The algorithm randomly chooses the leaders from the eligible set of agents. This process implements equal probability of selection for each eligible agent. Furthermore, an eligible agent may be selected multiple times. The proposed algorithm is more dependent on the choice of value for *γ*_*final*_ than *γ*_*initial*_. Higher values for *γ*_*initial*_ are likely to yield more exploration in the early phase, but degrade the overall convergence rate. *γ*_*final*_ value of zero indicates that only the best particle is eligible for selection. This strategy is similar to the common practice of reducing *kbest* to 1 in SGSA and similar algorithms. Implementation of the less stringent *γ*_*final*_ value of 0.1 in this study allows the proposed algorithm to outperform the SGSA and some of its variants.

However, varying *γ* from 1 to 0 is detrimental to the search process. This causes all agents, including agents with the worst performance, to be eligible for selection in the early phase. The algorithm can be misguided if the poorest performing agents are repeatedly selected, resulting in poor stability. Table 5 illustrates a performance comparison for different pairs of *γ*_*initial*_ and *γ*_*final*_. The algorithm was applied 30 times for each configuration to the multimodal function F6 to study the overall effects of *γ*. The worst performance is recorded for the extreme values of *γ*_*initial*_ = 1 and *γ*_*final*_ = 0. It is the least stable configuration as it exhibits the highest standard deviation. *γ*_*initial*_ = 0.9 and *γ*_*final*_ = 0.5 shows slightly better results. However the accuracy and stability of convergence are still unsatisfactory as indicated by the considerably high mean and standard deviation. The third case of *γ*_*initial*_ = 0.9 and *γ*_*final*_ = 0.1, in Table 5, can be considered adjustment from the first case results in. The algorithm is extremely stable and accurate in this configuration. The proposed algorithm exhibits similar behavior for the last two cases considered in Table 5, which illustrates that it is not too sensitive to the *γ* parameters provided they are within the general guidelines mentioned above.

An overall sensitivity analysis for *γ*_*initial*_ = 0.6 ± 0.1 and *γ*_*final*_ = 0.1 ± 0.1 on unimodal function F_3_, presented in Table 6, further illustrates the stable performance of the proposed algorithm. The last two rows in Table 6 show the cumulative effect of parameter value alterations. ‘Up’ row represents the results obtained from increasing both *γ* parameters. Conversely, ‘Down’ considers the scenario with both parameters reduced. Each configuration was implemented 30 times and the deviations are recorded as percentage of the results presented in Table 6, as shown in Eq (24).

$$Deviation(\%)=\frac{Solution(perturbed\;value)-Solution(original\;value)}{Solution(original\;value)}\times 100$$ (24)

**Table 6:** Results of the sensitivity analysis for minimization of F_3_with *t*_*max*_ = 500.

Parameter		Best (Deviation %)	Average (Deviation %)	Worst (Deviation %)	P-Value
*γ*_*initial*_ = 0.6	*γ*_*initial*_–*∆γ*_*initial*_ = 0.5	25.1129 (-0.3456)	26.81479 (0.7695)	29.2709 (0.6218)	0.157
*γ*_*initial*_ = 0.6	*γ*_*initial*_+*∆γ*_*initial*_ = 0.7	25.3654 (0.6563)	27.23589 (2.3520)	29.2769 (0.6424)	0.999
*γ*_*final*_ = 0.1	*γ*_*final*_–*∆γ*_*final*_ = 0	25.3758 (0.6976)	26.70139 (0.3434)	29.0484(-0.1430)	0.099
*γ*_*final*_ = 0.1	*γ*_*final*_+*∆γ*_*final*_ = 0.2	25.4258 (0.8960)	26.98846(1.4222)	29.1975(0.3695)	0.904
All	Down	25.4239 (0.8884)	26.63502 (0.0940)	29.2909 (0.6906)	0.001
All	Up	25.3027 (0.4075)	27.20617 (2.2403)	29.3829 (1.0068)	0.999

The results in Table 6 are attained using the recommended configuration of *γ*_*initial*_ = 0.6 and *Υ*_*final*_ = 0.1. The highest average performance deviation in Table 6 is 2.352% for increasing *γ*_*initial*_ by 0.1. This does not accumulate further as the ‘Up’ scenario shows deviation of 2.2403%. Thus, the performance degradation caused by *γ*_*initial*_ is partially mitigated by increasing *γ*_*final*_. It is evident from the p-values of Wilcoxon Rank Sum test that the recommended configuration achieves a statically significant performance improvement over only one of the six scenarios tested in Table 6. The ‘Down’ configuration resulted in p-value less than 0.05, which means the null hypothesis (no statistical difference) must be rejected and alternate hypothesis (recommended configuration outperforms ‘Down’ configuration) must be accepted. Thus, there is no statistically significant change in performance for most configurations of *γ*, indicating that the proposed algorithm is robust and the choice of values for *γ* is not critical.

The results presented in Tables 5 and 6 indicate that the proposed algorithm is applicable to both multimodal and unimodal functions. Overall, the analyses show that the proposed algorithm is sufficiently stable for application in real world optimization problems.

## Model Application

In the previous section, the robustness and effectiveness of the suggested algorithm (SL-GSA) have been tested through six benchmark functions. The performance of the proposed method to enhance signal to interference and noise ratio (*SINR*) of MVDR beamforming technique will be investigated and compared with conventional MVDR and MVDR-SGSA in this section. Two interference scenarios will be considered in this study. The first case is with two interference sources located at 30° and 50° while the second case is with four interference sources located at 30°, 50°, 25° and 60°. Both cases have one user at 0° and number of elements is four. The proposed algorithm was implemented by using MATLAB^®^.

### 7.1 Signal to Interference and Noise Ratio Calculation

In this paper, the SL-GSA was employed to improve the MVDR beamforming performance by increasing the *SINR* value of four element antenna array. The SL-GSA and SGSA were applied on MVDR technique to maximize the *SINR* by varying the complex weights.

In these algorithms, *w*_*mvdr*_ (MVDR weight vector) will be replaced as the first agent of initial population. The rest of the population is initialized randomly, as shown in Eq (26). This system will create a population of *N* agents to represent weight vectors. The weight vectors in every agent will contain *M* number of weights, where *M* is the number of elements in the array. The weights considered in the optimization process, thus, are represented as the matrix *W*_*NM*_.

$$x_{i}^{d}=w_{nm}$$ (25)

where dimension *d* is analogous to number of sensor *m*. Agent index, *i*, in SL-GSA and SGSA is set to *n*, as shown in Eq (26). The population weight vectors can be defined in the matrix format as follows:

$$W_{NM}=\left[\begin{array}{l} w_{mvdr1}\;w_{mvdr2}\;w_{mvdr3}\;w_{mvdr4}\;\\ w_{21}\;w_{22}\;w_{23}\;w_{2M}\\ w_{31}\;w_{32}\;w_{33}\;w_{3M}\\ .\;.\;.\;.\\ .\;.\;.\;.\\ w_{N1}\;w_{N2}\;w_{N3}\;w_{NM} \end{array}\right]$$ (26)

where *W*_*NM*_ is the weight vectors of *N* agents with *M* sensors and *W*_*mvdr*_ is the weight vectors from conventional MVDR beamformering. The fitness function employed in this study is the *SINR* as shown in Eq (27). Therefore, the optimization processes will try to maximize the fitness function by finding the corresponding optimal weight vectors.

$$FitnessFunction(F_{N})=\frac{P_{U}}{\sum_{i=1}^{I}P_{i}+N}$$ (27)

where *P*_*u*_ is the target user power, *P*_*i*_ is the power of interference at i interference, *I* is the number of interference sources and *N* is the noise.

### 7.2 Simulation

In this section, the validation of the proposed approach for real world applications with two interference scenarios is studied. The parameters of SGSA are chosen according to the recommendations and guidelines presented in literature [8]. These configurations of SGSA have also been used extensively after the development of SGSA [10–13]. The algorithms are simulated 20 times and the best results are recorded. The maximum iteration number and population size set to 100 and 50 respectively.

#### 7.2.1. Case 1: One user two interferences

Two interference sources at 30°, 50° and user at 0° have been assumed in the first case study.

**Fig 9:**
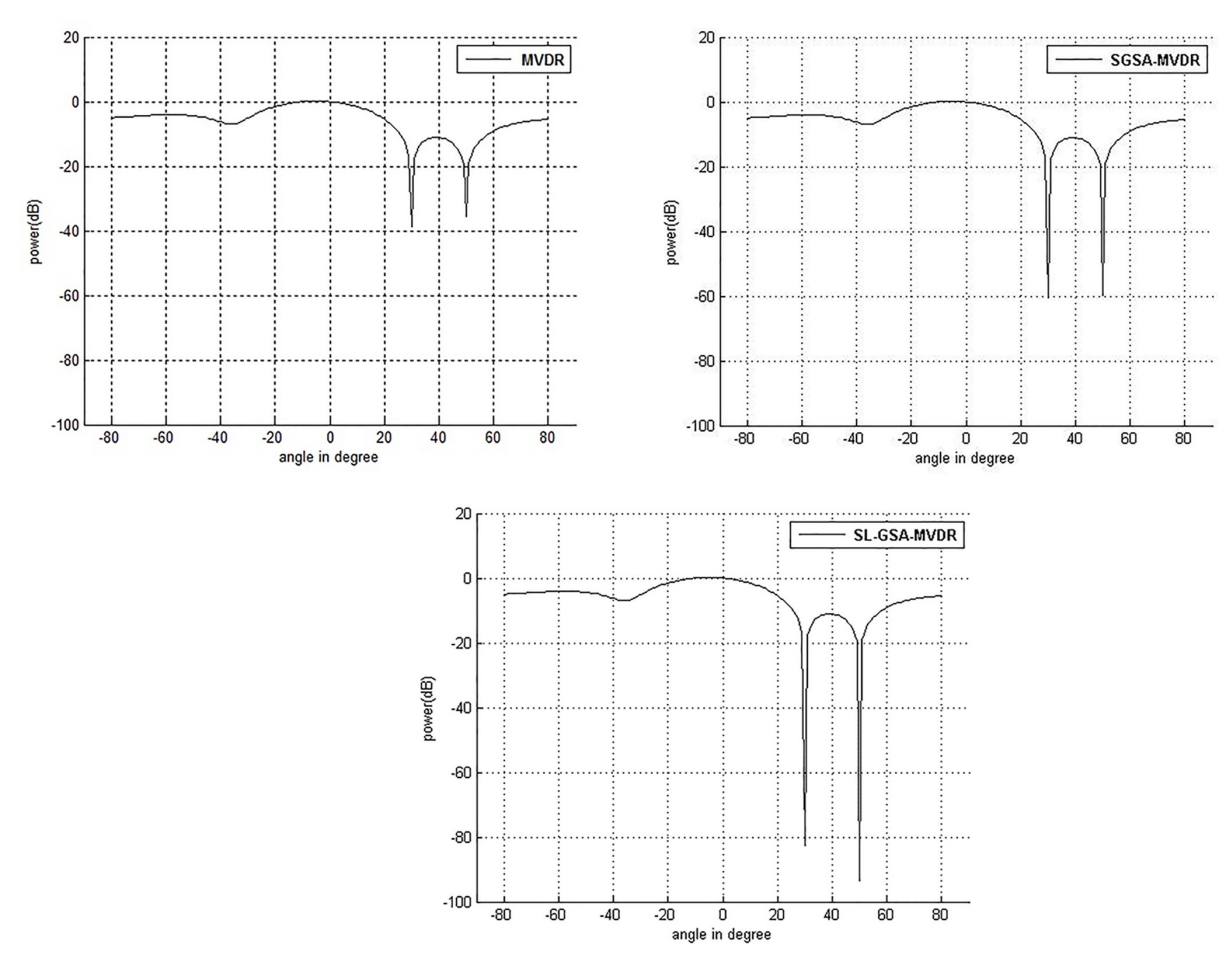
Comparison of performance of power response for user at 0° with two interferences at 30° and 50° with 100 iterations. (a) MVDR (b) SGSA-MVDR (c) SL-GSA-MVDR.

According to the weights in Table 7, the performance of power response for MVDR, SGSA-MVDR and SL-GSA-MVDR beamforming is plotted in Fig 9 to explain the target user and interference by using different values. Fig 9 clearly illustrates that the proposed algorithm achieves significantly deeper nulls than SGSA-MVDR and conventional MVDR.

**Table 7:** Comparison of weight vectors for conventional MVDR, SGSA-MVDR and SL-GSA-MVDR for user at 0° and interferences at 30° and 50°.

Name	MVDR	SGSA-MVDR	SL-GSA-MVDR
Weights	0.2233 + 0.1492i 0.3453–0.0650i 0.2387–0.003i 0.1925–0.0806i	0.2233 + 0.1491i 0.3453–0.0654i 0.2384–0.0034i 0.1927–0.0805i	0.2231 + 0.1491i 0.3454–0.0650i 0.2387–0.0035i 0.1926–0.0807i

In Table 8, 67.79% and 105.84% improvements of SINR are achieved by using SGSA and SL-GSA, respectively, over conventional MVDR. The superior performance of SL-GSA in this problem, along with the results of the benchmark problems, establishes SL-GSA’s as a suitable interference mitigation algorithm.

**Table 8:** Comparison of *SINR* calculation for conventional MVDR, SGSA-MVDR and SL-GSA-MVDR for user at 0° and interferences at 30° and 50°.

Name	SINR(dB)
MVDR	33.88
SGSA-MVDR	56.85
SL-GSA-MVDR	69.74

#### 7.2.2. Case 2: One user four interferences

Four interference sources at 30°, 50°, 25° and 60° and user at 0° have been assumed as the second case study.

**Fig 10:**
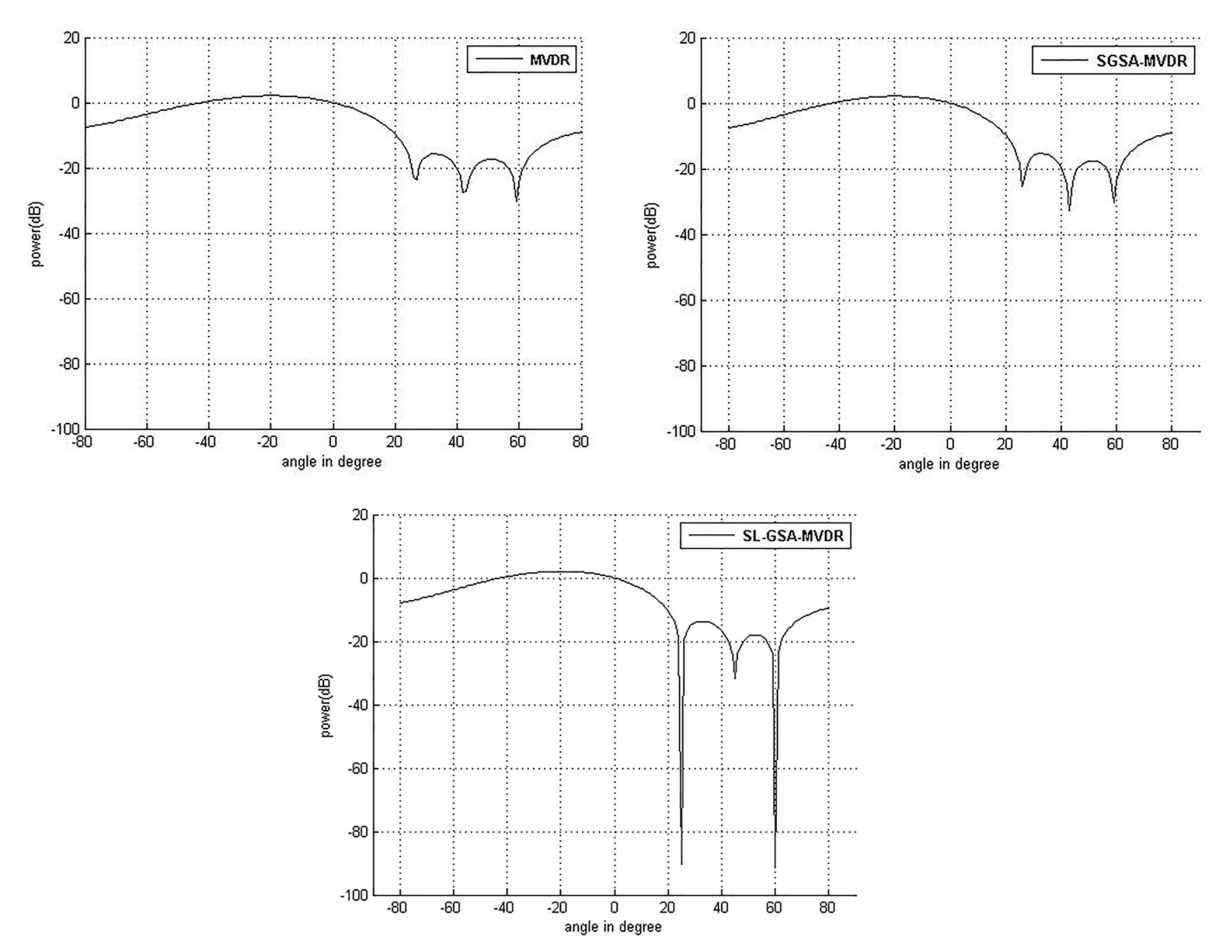
Comparison of performance of power response for user at 0° with interference at 30°, 50°, 25° and 60° with 100 iterations. (a) MVDR (b) SGSA-MVDR (c) SL-GSA-MVDR.

The power response for MVDR, SGSA-MVDR and SL-GSA-MVDR is plotted in Fig 10 according to the weights in Table 9 to explain the target user and interference by using different values. Conventional MVDR rarely manages to create sufficiently deep nulls towards more than 3 interference sources as shown in Fig 10. The two interferences at 25° and 30° produce one shallow null for MVDR and SGSA-MVDR. Furthermore, the nulls towards the other two interference sources are similarly shallow. Two of the three nulls created by MVDR are significantly improved by the implementation of the proposed algorithm. This clearly shows that SL-GSA can outperform the beamforming techniques in complex scenarios involving more than 3 interfering sources. The deep nulls correspond to superior *SINR* performance as shown in Table 10.

**Table 9:** Comparison of weight vectors for conventional MVDR, SGSA-MVDR and SL-GSA-MVDR for user at 0° and interferences at 30°, 50°, 25° and 60°.

Name	MVDR	SGSA-MVDR	SL-GSA-MVDR
Weights	-0.0067 + 0.2276i 0.5071 + 0.3017i 0.5065–0.3022i 0.0069–0.2271i	-0.0059 + 0.2281i 0.5061 + 0.3012i 0.5059–0.3022i 0.0058–0.2271i	-0.0029 + 0.2297i 0.4973 + 0.3001i 0.5008–0.2957i 0.0009–0.2310i

**Table 10:** Comparison of *SINR* calculation for conventional MVDR, SGSA-MVDR and SL-GSA-MVDR for user at 0° and interference at 30°, 50°, 25° and 60°.

Name	SINR(dB)
MVDR	12.17
SGSA-MVDR	12.21
SL-GSA-MVDR	12.76

Table 10 illustrates the improvement of *SINR* that are 0.32% and 4.84% by SGSA and SL-GSA, respectively, as compared to conventional MVDR method. This case represents the most complex interference mitigation optimization problem in this paper. The results are consistent with the previous case, indicating SL-GSA to be generally superior for real world optimization problems compared to SGSA.

Referring to the both simulation cases, when the interference sources become closer, it is more difficult for both conventional MVDR and artificial intelligence based technique to enhance null level at the interference direction. From the simulation result, it is clearly observed that, SGSA performs inferior than SL-GSA. The superior exploratory and exploitive properties of SL-GSA over SGSA have resulted in better beam steering and interference mitigation performances in both cases.

## Conclusions

In this paper, a stochastic leader gravitational search algorithm via randomizing the choice of *kbest* utilized in total force calculation of the original algorithm was proposed. The use of *kbest* in SGSA that allows the heaviest few agents to apply force on other agents may often causes premature convergence due to one or a group of dominant agents. The proposed SL-GSA alleviates this problem while simultaneously enhancing the exploration in the early phase of optimization by randomly selecting the leading agents. This stochastic process prevents the aforementioned domination and results in superior performance for both benchmark functions and real world optimization problems. The parameter *γ* provides an elegant method for balancing the exploration and exploitation properties of the proposed algorithm. Overall, SL-GSA exhibits higher robustness, convergence rate, accuracy, and stability without increasing computational difficulty in comparison with SGSA. The achievement of satisfactorily deep nulls in complex interference environments indicates that the proposed algorithm is ready to be implemented in real world applications.

## References

[1] Capon J (1969) High-resolution frequency-wavenumber spectrum analysis. Institute of Electrical and Electronics Engineers (IEEE) 57 (8): 1408–1418.

[2] Dahrouj H, Yu W (2010) Coordinated beamforming for the multicell multi-antenna wireless system. IEEE transactions on wireless communications 9(5): 1748–1759.

[3] Tiong SK, Salem B, Koh SP, Sankar KP, Darzi S (2014) Minimum variance distortionless response (MVDR) beamformer with enhanced nulling level control via dynamic mutated artificial immune system. The Scientific World Journal, Article ID 164053, 10 pages. 10.1155/2014/164053 PMC4070486 25003136

[4] Darzi S, Tiong SK, Islam MT, Ismail M, Kibria S, Salem B (2014) Null steering of adaptive beamforming using linear constraint minimum variance assisted by particle swarm optimization, dynamic mutated artificial immune system, and gravitational search algorithm. The Scientific World Journal, Article ID 724639, 10 pages. 10.1155/2014/724639 PMC4132335 25147859

[5] Khodier MM, Christodoulou CG (2005) Linear array geometry synthesis with minimum side lobe level and null control using particle swarm optimization. IEEE Transactions on Antennas and Propagation 53(8): 2674–2679.

[6] Zaharis ZD, Yioultsis TV (2011) A novel adaptive beamforming technique applied on linear antenna arrays using adaptive mutated Boolean PSO. Electromagnetic Research, 117: 165–179.

[7] Kaid OO, Debbat F, Boudghene SA (2012) Null steering beamforming using hybrid algorithm based on honey bees mating optimization and tabu search in adaptive antenna array. Electromagnetic Research C, 32: 65–80.

[8] Rashedi E, Nezamabadi-pour H, Saryazdi S (2009) GSA: gravitational search algorithm, Information Sciences, 179: 2232–2248.

[9] Liu FL, Wang JK (2011) Robust mvdr beamformer for nulling level control via multi-parametric quadratic programming. Electromagnetics Research C, 20: 239–254.

[10] Khajehzadeh M, Taha MR, Eslami M (2013) Efficient gravitational search algorithm for optimum design of retaining walls. Structural Engineering and Mechanics 45(1):111–127.

[11] Khajehzadeh M, Taha MR, Shafie AE, Eslami M (2012) A modified gravitational search algorithm for slope stability analysis. Engineering Applications of Artificial Intelligence 25:1589–1597.

[12] Binjie G, Feng P (2013) Modified gravitational search algorithm with particle memory ability and its application. International Journal of Innovative Computing, Information and Control 9(11):4531–4544.

[13] Rashedi E., Nezamabadi-pour H., Saryazdi S., 2010 BGSA: binary gravitational search algorithm, Natural Computing, 9(3), 727–745.

